# Combining Mesoporosity and Shape Selectivity in FAU–MFI Interzeolite Intermediates for Renewable Xylenes Production

**DOI:** 10.1021/acssuschemeng.5c05295

**Published:** 2025-08-19

**Authors:** Daniela O. Campos, Anna D. F. F. Monsores, Natasha M. Suguihiro, Donato A. G. Aranda, Javier Garcia Martinez, Pedro N. Romano, João M.A.R. de Almeida

**Affiliations:** † Escola de Química, 28125 Universidade Federal do Rio de Janeiro, Av. Athos da Silveira Ramos, 149, Rio de Janeiro 21941-909, Brazil; ‡ LIPCAT/IDlab (Laboratório de Intensificação de Processos e Catálise), Universidade Federal do Rio de Janeiro (UFRJ), Rio de Janeiro 21941-594, RJ, Brazil; § Laboratorio de Nanotecnología Molecular, Departamento de Química Inorgánica, 16718 Universidad de Alicante, 03690 Alicante, Spain; ∥ Nanotechnology Engineering Program, Alberto Luiz Coimbra Institute for Graduate Studies and Research in Engineering (COPPE), Federal University of Rio de Janeiro, Avenida Horacio Macedo, 2030, Rio de Janeiro 21941-972, RJ, Brazil; ⊥ Campus Duque de Caxias, Universidade Federal do Rio de Janeiro, Rodovia Washington Luiz, 19593, Rio de Janeiro 25240-005, Brazil; # Instituto de Química, Universidade Federal do Rio de Janeiro, Av. Athos da Silveira Ramos, 149, Rio de Janeiro 21941-909, Brazil

**Keywords:** hybrid zeolites, shape selectivity, interzeolite transformation intermediates, hierarchical porosity, microwave, biomass valorization, xylene synthesis

## Abstract

Interzeolite Transformation Intermediates (ITIs) were synthesized via microwave-assisted conversion of FAU to MFI phases and applied in the transformation of 2,5-dimethylfuran (DMF) and ethanol into xylenes. Structural, textural, and acidic properties were characterized by X-ray diffraction (XRD), scanning electron microscopy (SEM), transmission electron microscopy (TEM), NMR, Raman, and NH_3_-TPD. Catalytic tests, including Friedel–Crafts alkylation and triisopropylbenzene cracking, revealed that hybridization enhanced molecular accessibility and acid site distribution. The optimized material, HyZ-38, which preserved features of both FAU and MFI, achieved a high xylene yield of 45.2%. This performance was linked to the balance between mesoporosity and shape selectivity, enabling better diffusion and fewer side reactions. Additionally, microwave-assisted synthesis reduced both time and energy consumption. These results underscore the potential of interzeolite transformation as a sustainable strategy for designing efficient catalysts for biomass valorization and renewable aromatic production under demanding reaction conditions.

## Graphical abstract

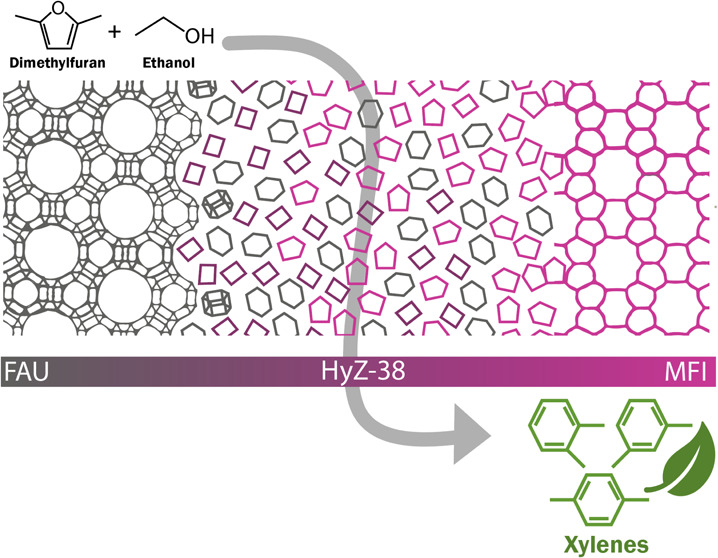

## 1 Introduction

The increasing demand for versatile, durable, and cost-effective materials has made solidified polymers indispensable components of modern life.^1^ Poly­(ethylene terephthalate) (PET) is one of the most widely produced and consumed polymers globally, with broad applications in the packaging, textile, and film industries.^2^ Its advantageous physicochemical properties, including mechanical strength, thermal stability, and recyclability, make PET a strategic material in multiple industrial sectors.^3–5^

Nevertheless, the PET production chain remains heavily reliant on fossil-fuel-based resources. Traditionally, PET has been synthesized via the polycondensation of monoethylene glycol (MEG) and terephthalic acid (PTA), which is obtained through the oxidation of *p*-xylene, an aromatic compound directly derived from petroleum.^6–9^ This conventional route not only demands harsh reaction conditions and nonrenewable feedstocks but is also linked to substantial greenhouse gas emissions and the generation of toxic waste.^10,11^ This scenario contrasts with the principles of green chemistry and international sustainability commitments in the context of global energy transition and industrial decarbonization.^12–14^

Within this context, the development of alternative pathways for PET production based on renewable feedstocks and cleaner processes has shifted from merely optional to strategic imperative.^15^ Among the most promising strategies is the synthesis of renewable xylenes via a reaction between 2,5-dimethylfuran (DMF) and ethanol.^16–19^ DMF can be derived from sugars present in lignocellulosic biomass,^20,21^ whereas ethanol is widely produced by fermentation of various plant-based sources.^22^ The reaction between these two compounds leads to the formation of xylenes, which can subsequently be converted into *p*-xylene and then oxidized to PTA. This route constitutes a green platform for PET production, integrating two distinct renewable sources and promoting the valorization of biomass residues.

The success of this strategy relies significantly on the use of efficient solid catalysts. Zeolites have emerged as natural candidates for this application,^23,24^ particularly MFI zeolites (e.g., ZSM-5), which are widely recognized for their tunable acidity, thermal stability, and porous architecture conducive to xylenes formation.^25–27^ However, the direct application of these zeolites in reactions involving biomass-derived molecules such as DMF encounters a fundamental challenge: the severe diffusional limitations imposed by their intrinsic microporosity.^28,29^ Bulky functionalized molecules often struggle to reach the active sites within the zeolite framework, which can hinder the overall catalytic performance, even when the intrinsic selectivity remains high.

The introduction of mesoporosity into zeolites has proven to be an effective strategy to overcome diffusion limitations and improve catalytic performance in xylenes production.^30,31^ In particular, hierarchical MFI-type zeolites, combining micro- and mesoporosity, have been extensively explored to enhance mass transport and catalyst efficiency in biomass-derived aromatic production. Kim et al. reported significant improvements in xylenes yield using MFI nanosheet zeolites due to better reactant diffusion and external acid site accessibility. Likewise, McGlone et al. showed that simple desilication of ZSM-5 increases mesoporosity, facilitating diffusion and improving conversion and selectivity in renewable aromatic synthesis.

Another even more promising and innovative strategy to overcome these structural limitations involves the interconversion of FAU zeolite (Y-type structure) into MFI, leading to the formation of hierarchical hybrid materials.^28^ This transformation enables the integration of the wide accessibility inherent to the FAU framework with the intrinsic selectivity of MFI, whose network of intersecting microporous channels promotes the formation of light aromatic compounds such as xylenes.^32^ In addition to yielding an intermediate morphology that retains the structural features of both phases,^33^ this approach produces hierarchical catalysts with a more efficient distribution of active sites, which are key attributes for converting bulky biomass-derived molecules.^29^ Moreover, performing this interconversion under microwave assistance significantly enhances the synthesis rate and energy efficiency,^31^ while enabling the electrification of the catalyst preparation process, thus contributing to the more sustainable development of catalysts suited to the needs of the modern chemical industry.^34^

In this context, the present study focuses on investigating the potential of hybrid zeolites obtained through microwave-assisted FAU-to-MFI interzeolite transformation as catalysts in a sustainable catalytic reaction of high societal relevance: the production of xylenes from 2,5-dimethylfuran (DMF) and ethanol, both derived from biomass. This route enables the integration of renewable feedstocks into the production of high-value aromatics with broad industrial applications. The proposed approach combines key principles of catalysis, green chemistry, and process intensification, aiming to demonstrate how underexplored zeolitic materials can contribute to the development of cleaner and more sustainable solutions for the chemical industry.

## 2 Materials and Methods

### 2.1 Materials

The reagents employed in this work included the following: high-purity deionized water (resistivity of 18.2 MΩ·cm, Millipore, Billerica, MA); ammonium chloride (NH_4_Cl, analytical grade, Isofar); commercial MFI zeolite CBV 8014 (Zeolyst International, SiO_2_/Al_2_O_3_ ratio = 80); commercial FAU zeolite CBV 780 (Zeolyst International, SiO_2_/Al_2_O_3_ ratio = 80); sodium hydroxide (NaOH, analytical grade, Synth); tri-*n*-propylamine (98%, Sigma-Aldrich); 1-bromohexadecane (97%, Sigma-Aldrich); triisopropylbenzene (TiPBz, Sigma-Aldrich); methanol (99.99%, Sigma-Aldrich); acetonitrile (99.9%, Sigma-Aldrich); diethyl ether (99.9%, Sigma-Aldrich); mesitylene (98%, Sigma-Aldrich); benzyl alcohol (98%, Sigma-Aldrich); ethanol (99.5%, Isofar); *n*-heptane (99.99%, Sigma-Aldrich); and 2,5-dimethylfuran (99%, Sigma-Aldrich).

### 2.2 Synthesis of Cetyltripropylammonium Bromide (CTPABr)

Cetyltripropylammonium bromide (CTPABr, acting as SDA and porogen) was synthesized via the reaction of triethylamine (4.76 mL) with 1-bromohexadecane (9.17 mL). The reagents were added to a round-bottom flask containing 30% methanol/acetonitrile solvent mixture. The resulting solution was refluxed at 80 °C for 48 h. After the completion of the reaction, the solvent was removed using a vacuum rotary evaporator. The crude product was purified by recrystallization and repeated three times in a methanol/diethyl ether mixture (2:10) to yield a pure compound.^28^

### 2.3 FAU to MFI Interconversion

The synthesis procedure, adapted from the method reported by Mendoza-Castro et al.,^28^ involved the conversion of FAU zeolite (CBV780) into the MFI phase using 2 g of FAU, 0.75 g of CTPABr, and 57 mL of 0.08 M aqueous NaOH solution. Initially, the mixture was aged at room temperature under continuous stirring for 1 h. The resulting suspension was transferred to a Teflon-lined vessel and subjected to thermal treatment in a FlexiWave microwave reactor. The process was conducted at 150 °C with a power input of 1500 W for 24, 38, 48, and 60 h, enabling the investigation of the structural evolution throughout the interconversion process.

Following synthesis, the resulting materials were dried in an oven at 100 °C for 12 h to remove residual moisture. Subsequently, calcination was performed under an air atmosphere by gradually increasing the temperature at a rate of 5 °C/min until reaching 550 °C, which was maintained for 3 h to ensure complete removal of the organic template (CTPABr).

After calcination, the material underwent ion exchange using a 1 M NH_4_Cl solution for 24 h at 80 °C, followed by a second calcination under identical conditions to convert the material into its acidic form, thereby promoting the formation of Brønsted acid sites in the final product.

### 2.4 Characterization Methods

The crystalline structures of the materials were characterized by powder X-ray diffraction (XRD) using a Rigaku Miniflex II diffractometer with Cu–Kα radiation. Measurements were carried from 5 to 55° 2θ, and the CVBV8014 commercial MFI zeolite used as a reference.

The morphology and structural features of the particles were examined by scanning electron microscopy (SEM) using a JEOL JSM-IT700HR equipped with an energy-dispersive X-ray spectroscopy (EDS) detector. For more detailed insights into the internal structure of the materials, transmission electron microscopy (TEM) was performed using a JEOL JEM-2100F instrument.

The textural properties of the samples were investigated by argon 87K physisorption using an Autosorb iQ analyzer (Anton-Paar, Graz, Austria). Before the measurements, the samples were degassed at 350 °C for 9 h under high vacuum. The specific surface area was determined using the Brunauer–Emmett–Teller (BET) method, whereas the micropore and mesopore volumes were estimated using the NL-DFT model.

The acidity of the zeolites was assessed by ammonia temperature-programmed desorption (NH_3_-TPD) using a Micromeritics AutoChem II analyzer equipped with a thermal conductivity detector (TCD). samples (100 mg) were pretreated at 300 °C for 1 h under a continuous helium flow (25 mL/min). Subsequently, ammonia adsorption (15% NH_3_ in He) was performed at 150 °C for 1 h, and desorption was monitored as the temperature was ramped at 10 °C/min up to 500 °C.

The chemical composition of the materials was determined using atomic absorption spectroscopy (AAS) to quantify the Si/Al molar ratio. Analyses were conducted using an AA-700 atomic absorption spectrophotometer (Shimadzu, Kyoto, Japan) following an acid-digestion procedure assisted by microwave heating. Approximately 500 mg of the sample was treated with a mixture of 4 mL nitric acid (HNO_3_), 12 mL hydrochloric acid (HCl), and 1 mL hydrofluoric acid using a Milestone Ethos X Advanced Microwave Extraction system (Metrohm, Herisau, Switzerland). The digestion process was carried out in two stages: initially at 170 °C for 5 min, followed by heating at 200 °C for 25 min. After cooling to room temperature, the mixture was neutralized with boric acid to form complex residual fluoride ions and then subjected to a second microwave heating cycle under the same conditions to ensure complete digestion. The resulting solution was filtered to remove particulates and diluted with ultrapure water for subsequent analyses.

Structural analysis of the zeolite framework was performed by magic-angle spinning nuclear magnetic resonance (MAS NMR). Measurements were carried out on a Bruker Avance III 400WB spectrometer operating at 104.23 MHz for ^27^Al (single-pulse excitation, 12 kHz spinning rate) and at 79.46 MHz for ^29^Si (single-pulse excitation, 5 kHz spinning rate).

Finally, the molecular structures of the materials were characterized using Raman spectroscopy. Spectra were acquired using a dispersive Xplora/Horiba system equipped with a 532 nm laser source.

### 2.5 Catalytic Assessment

#### 2.5.1 Friedel–Crafts Alkylation (FC)

Friedel–Crafts alkylation of mesitylene (ME) with benzyl alcohol (BA) was carried out following the protocol established by Jain et al.^35^ Each experiment used 95 mmol of mesitylene, 1 mmol of benzyl alcohol, and 100 mg of the catalyst.

Reactions were performed in a sealed round-bottom flask maintained at 120 °C under continuous stirring to ensure homogeneous mixing of the reactants. Reaction mixture samples were withdrawn at regular intervals and analyzed by gas chromatography with flame ionization detection (GC-FID) using a Shimadzu instrument (column DB—1, 0.25 mm, 100 m), enabling quantification of benzyl alcohol conversion and determination of selectivity toward 1,3,5-trimethyl-2-benzylbenzene (TM2B) and dibenzyl ether (DBE).

The turnover frequency (TOF) was determined according to the following equations

$$TOF\;\left(h^{-1}\right)=\left(N_{BA,0}-N_{BA}\right)/\left(\mathrm{tx}N_{NH_{3}}\right)$$

Where *N*_BA0_ denotes the initial number of moles of BA, and *N*_BA0_ corresponds to the final number of moles of BA. The *N*_NH_3__ represents the number of moles of acid sites, determined by NH_3_-TPD.

#### 2.5.2 Catalytic Cracking of 1,3,5-Triisopropylbenzene (TiPBz)

The catalytic cracking of 1,3,5-triisopropylbenzene (TiPBz) was carried out following the protocol described by Abdulridha et al.^36^ For this procedure, 15 mg of zeolite were loaded into a borosilicate glass tube (Restek 20793) with an inner diameter of 4 mm, outer diameter of 6.3 mm, and length of 72 mm positioned between two layers of quartz wool (Shimadzu). Before the reaction, the zeolites were dried overnight to eliminate residual moisture.

In each experiment, 2 μL of TiPBz were injected into the system, vaporized, and carried to the catalytic bed by a helium stream at 200 mL·min^–1^. The injector temperature was maintained at 350 °C to ensure complete vaporization of the reagent. The resulting products were partially directed to a capillary column (DB-WAX, 0.20 mm i.d., 50 m length, 0.2 μm film thickness, Agilent) at a split ratio of 200:1. The products were then analyzed using a flame ionization detector (FID) operated at 300 °C. The column temperature program was initiated at 80 °C and ramped at 10 °C·min^–1^ up to 220 °C, where it was held for 10 min. Each experiment was performed 20 times per sample using the pulse injection technique on a GC Thermo Trace 1610 system to ensure the reproducibility and robustness of the collected data.

The turnover frequency (TOF) was determined according to the following equations

$$TOF\;\left(h^{-1}\right)=\left(N_{\mathrm{TiPBz},0}-N_{\mathrm{TiPBz}}\right)/\left(\mathrm{tx}N_{NH_{3}}\right)$$

Where *N*_TiPBz,0_ denotes the initial number of moles of TiPBz, and *N*_TiPBz_ corresponds to the final number of moles of TiPBz. The *N*_NH_3__ represents the number of moles of acid sites, determined by NH_3_-TPD.

#### 2.5.3 Diels–Alder Cycloaddition of 2,5-DMF and Ethanol to Xylenes

The Diels–Alder cycloaddition (DAC) reaction between dimethylfuran (DMF) and ethanol for xylene production was performed in a 50 mL batch reactor, initially pressurized to 20 bar with N_2_ and maintained under continuous stirring at 900 rpm. A total of 13.8 mL of a reaction mixture containing DMF, ethanol, and *n*-heptane in a molar ratio of 1:1:4 was added to the reactor with a catalyst mass normalized to the total amount of acid sites, based on the HyZ-38 hybrid (0.15 g). The reaction time was initiated once the target temperature of 300 °C was reached.^16,17^ After 12 h, the reaction was quenched by shutting the heating system off and cooling the reactor in an ice bath. The reactor was then depressurized in a controlled manner. Liquid samples were collected, filtered through 0.22 μm membranes, and analyzed using a Shimadzu gas chromatograph (GC-2030) equipped with an RTX-1 capillary column (100 m × 0.25 μm × 0.25 mm) and a flame ionization detector (FID). The reactants and products were quantified using response factor calibration based on external standards, including DMF, xylene mixture, and ethanol.

Chromatographic peaks were identified by comparing their retention times with those of chemical standards and related compounds. Product identification was further confirmed by gas chromatography–mass spectrometry (GC-MS), which enabled detailed compound identification based on spectral library matching.

The conversion of DMF, xylene yield, and selectivity were calculated using the following equations,^37,38^ respectively

$$X_{\mathrm{DMF}}\;\left(\%\right)=\left(\left(N_{\mathrm{DMF},0}-N_{\mathrm{DMF}}\right)/N_{\mathrm{DMF},0}\right)\times 100$$

$$Y_{\mathrm{xylenes}}\;\left(\%\right)=\left(\left(N_{\mathrm{xylenes}}\right)/\left(N_{\mathrm{DMF},0}\right)\right)\times 100$$

$$S_{\mathrm{xylenes}}\;\left(\%\right)=\left(\left(N_{\mathrm{xylenes}}\right)/\left(N_{\mathrm{DMF},0}-N_{\mathrm{DMF}}\right)\right)\times 100$$

Where *N*_DMF,0_ denotes the initial number of moles of DMF, and *N*_DMF_ corresponds to the final number of moles of DMF. The variable *N*_xylenes_ refers to the final number of moles of xylenes. *N*_NH_3__ represents the number of moles of acid sites, determined by NH_3_-TPD.

## 3 Results

### 3.1 Synthesis and Characterization

The commercial FAU zeolite (CBV780) was subjected to hydrothermal treatment in an alkaline solution containing CTPABr under microwave heating at 150 °C for different periods of time, in order to obtain a series of FAU-to-MFI interzeolite transformation intermediates (ITIs). Structural evolution was monitored by powder X-ray diffraction (XRD, Figure 1a). After 24 h of synthesis, the materials were X-ray amorphous, showing no characteristic diffraction peaks of either the parent zeolite (FAU) or target MFI phase.^29,39^ After 48 h, characteristic diffraction peaks of ZSM-5 (7.9, 8.9, 23.1, 23.4, and 24.0°, in accordance with JCPDS-44–0003) were observed, with no remaining traces of the original FAU structure, indicating progressive conversion to the MFI phase.^29,39^ As the synthesis time increased, the diffraction peak intensities also rose, indicating the development of crystallinity related to the formation of the MFI zeolite. After 60 h, fully crystalline ZSM-5 was obtained, free of secondary phases or amorphous silica.^28,33,34^

**Figure 1:**
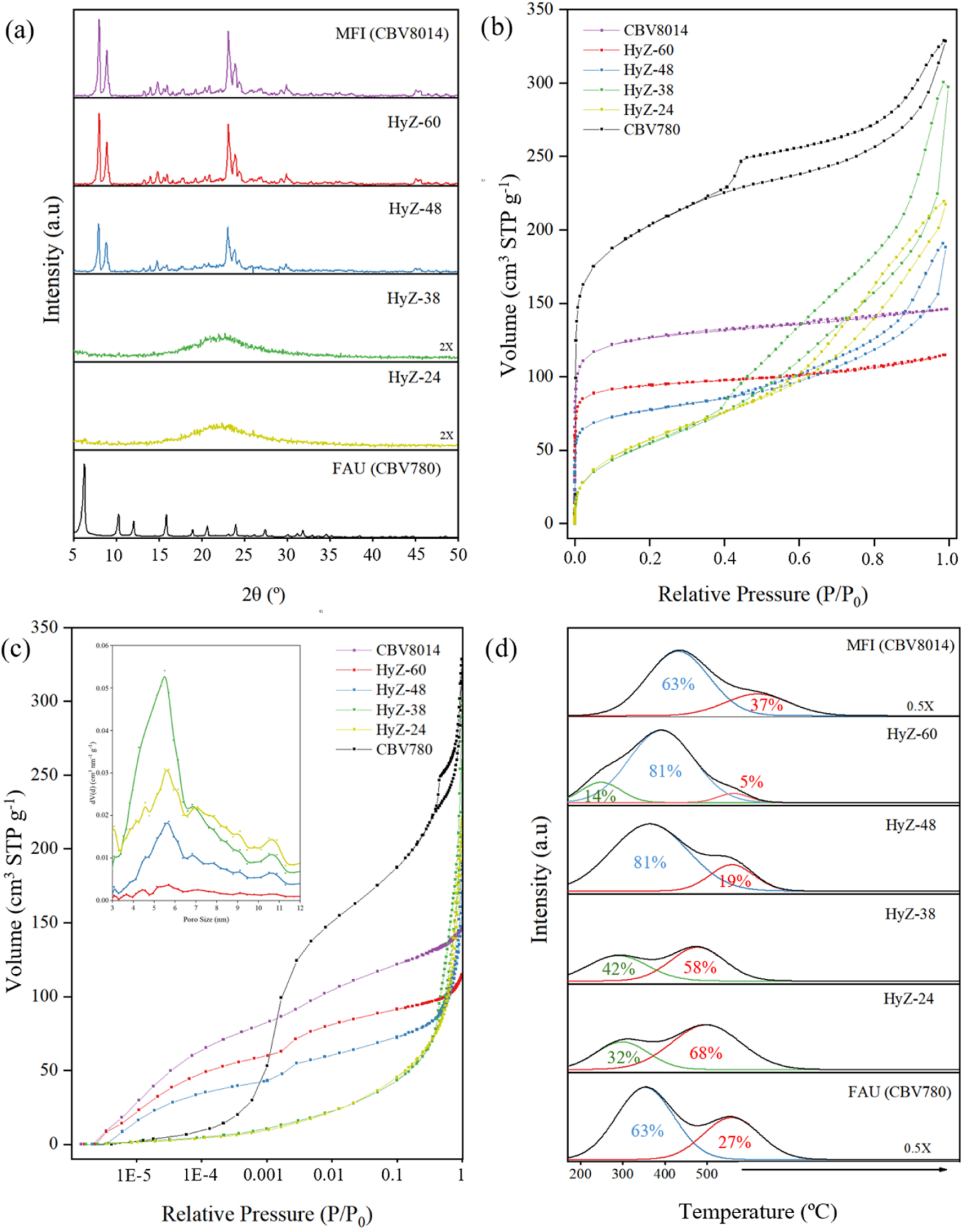
Characterizations of the catalysts: (a) XRD patterns, (b) Ar physisorption at 87 K, (c) Ar physisorption plotted in semilogarithmic scale and the corresponding pore size distribution calculated by NL-DFT from the isotherms and (d) NH_3_-TPD (total acidity).

The evolution of textural properties of the samples obtained during the interzeolite conversion process was monitored by argon physisorption at 87 K (Figure 1b). The parent FAU zeolite (CBV780) exhibits a type I isotherm, characteristic of microporous materials, with pronounced argon uptake at low relative pressures (*P*/*P*_0_ < 0.1).^40^ However, some mesoporosity is also present, featuring a hysteresis loop and cavitation, caused by the ultrastabilization treatment, i.e., a steam-assisted partial dealumination.^41^

The ITI samples obtained after 24 h (HyZ-24) and 38 h (HyZ-38) exhibited mixed type IV isotherms, indicating that at short interconversion times, only mesoporosity is observed (Figure 1b,c and Table 1), which is consistent with the lack of any zeolite crystallinity (Figure 1a).^40^ The sharp increase in adsorption at *P*/*P*_0_ ≈ 0.3 suggests the presence of well-defined mesopores centered around 4 nm (Figure 1c). The use of CTPABr, an organic structure-directing agent (OSDA) that combines the structure-directing capability of quaternary amines (tripropylcetyl) with the micelle-forming properties of long-chain alkyl groups, promotes the selective formation of the MFI phase while simultaneously facilitating the development of well-defined mesoporosity.^28^

**Table 1:** Textural and Acidic Properties of the Samples

catalyst	Si/Al^a^	*S*_BET_ (m^2^/gzeo)^b^	*V*_micro_ (cm^3^/gzeo)^c^	*V*_meso_ (cm^3^/gzeo)^c^	total acidity (μmol/gzeo)^d^
CBV780	52	618	0.25	0.15	523
HyZ-24	44	174	<0.01	0.23	264
HyZ-38	47	182	0.01	0.24	262
HyZ-48	48	246	0.11	0.10	298
HyZ-60	49	317	0.16	0.03	347
CBV8014	40	413	0.20	0.04	421

a Bulk Si/Al obtained through atomic absorption spectrometry.

b Specific surface area calculated using the BET method.

c Volume of micropores and mesopores calculated using the NL-DFT model.

d Total acidity was calculated using NH_3_-TPD.

As the synthesis time was extended to 48 h (HyZ-48) and 60 h (HyZ-60), a gradual transition in the adsorption isotherm profile was observed, shifting from a mixed type I + IV (HyZ-48) behavior to a predominantly type I (HyZ-60) profile, which is characteristic of microporous materials. This result confirms the structural conversion progress from FAU to the MFI phase, in which a reduction in mesopore volume accompanies a more defined crystal growth. This decrease in mesoporosity is attributed to the internal framework reorganization and consolidation of a denser microporous network, typical of the MFI structure. Previous studies have also associated this reduction in mesoporosity with the possible degradation of CTPA^+^ molecules via Hofmann elimination, favored under the hydrothermal and alkaline conditions of the medium, resulting in surfactant decomposition and hindering the maintenance of mesoporosity in samples subjected to longer synthesis times.^28,42^

As a reference, a commercial MFI zeolite (CBV8014) was analyzed for comparison. Its isotherm exhibits a profile similar to that of samples synthesized for longer durations. However, a slight residual mesoporosity was observed, possibly resulting from the postsynthesis treatments applied during industrial production.

The gradual transformation of FAU into MFI zeolite is clearly reflected in the evolution of the isotherms at low relative pressures, as shown on the logarithmic scale (starting from *P*/*P*_0_ = 10^–7^) (Figure 1c), revealing a clear transition in the adsorption profile with an increase in treatment time. FAU and MFI zeolites exhibit markedly different adsorption behaviors in the low relative pressure range (*P*/*P*_0_ < 10^–4^), which is consistent with their distinct microporous structures. The intermediate samples (HyZ-24 to HyZ-60) revealed a continuous evolution between these two extremes, with a progressive decrease in mesoporosity and an increase in microporosity, confirming the reorganization of the porous network during the interzeolite transformation.^28^

A key feature of any solid catalyst for the conversion of DMF and ethanol into xylenes is strong acidity. For this reason, the acidity of all materials was assessed by NH_3_-TPD (Figure 1d). This technique allows for the identification of the strength of acid sites present in the samples, distinguishing weak (200–350 °C), moderate (350–450 °C), and strong sites (>450 °C).^43^ What stands out in the results is how this distribution changes as the zeolite evolves.

In the early stages of interconversion (HyZ-24 and HyZ-38), weak and strong acid sites were predominant, whereas moderate sites were barely present (see Figure 1d). This behavior may be correlated with the amorphous nature of the early intermediate samples, which do not yet possess a well-defined crystalline structure and contain a significant amount of extra-framework aluminum, as confirmed later by the ^27^Al NMR results.

Starting at 48 h of treatment, the NH_3_-TPD profiles (Figure 1b) revealed the emergence of moderate acidic sites (350–450 °C), indicating a significant shift in the acidity profile of the materials. This transition is related to the gradual reintegration of extra-framework aluminum into the zeolite structure, as will be demonstrated throughout the manuscript by the ^27^Al NMR analyses. Restoration of the crystalline structure promoted the formation of new acid sites and contributed to both an increase in the total acidity and a noticeable change in the acid strength of the HyZ-48 and HyZ-60 samples.

These NH_3_-TPD results highlight that the controlled interconversion from FAU to MFI allows for the modulation of the acid site distribution and strength. In the early stages of transformation, the coexistence of weak and strong acid sites (Figure 1d) can be particularly beneficial for tandem reactions, such as the conversion of ethanol and 2,5-dimethylfuran (DMF) into xylenes.^20^ While strong acid sites activate the reactant molecules and drive the conversion process, weak acid sites help suppress undesired side reactions, thereby enhancing selectivity toward the formation of the desired products.

The total Si/Al ratio (SAR) was determined using Atomic Absorption Spectroscopy (AAS), a technique that enables precise quantification of the chemical Si/Al ratio in the synthesized materials (Table 1). The parent zeolite exhibited a total SAR of 54, which was higher than the value reported by the supplier (Si/Al = 40). To validate these results, the zeolite unit cell was determined, and the Si/Al ratio was calculated according to the method established by ASTM standard D3942–19.^44^ This resulted in a value of 52, thus reinforcing the reliability of the AAS measurements. The synthesized materials exhibited a total SAR similar to that of the commercial zeolite CBV780 (Si/Al = 52). This correlation may be related to the ability of quaternary amines to minimize silica dissolution in basic media, thereby contributing to the preservation of high Si/Al ratios.^28^

The morphology and structure of the materials undergo significant changes during interzeolite transformation. To monitor this evolution, scanning electron microscopy (SEM) and transmission electron microscopy (TEM) analyses were performed. As shown in Figure 2a, the SEM micrographs revealed that the parent FAU zeolite consisted of well-defined crystals with dimensions below 1 μm.^45^ As shown in Figure 2b,c, corresponding to the initial stages of the interconversion process, irregular aggregates and particles with reduced crystalline phases were observed, suggesting the coexistence of amorphous or semicrystalline phases. This evolution is supported by the X-ray diffraction (XRD) results (Figure 1), which reveal the loss of the initial crystalline structure and the gradual appearance of characteristic MFI peaks as the interzeolite process progressed. In the final stages (Figure 2d,e), the particle morphology becomes more organized, exhibiting pillar-like prismatic structures with a slight resemblance to the typical morphology of MFI zeolites synthesized by other methods reported in the literature,^39^ confirming the successful structural conversion. Additionally, SEM analysis was performed on the commercial MFI zeolite (CBV8014). However, the obtained image revealed an irregular morphology, not characteristic of MFI, possibly resulting from some treatment or stabilization process to which the commercial sample may have been subjected.

**Figure 2:**
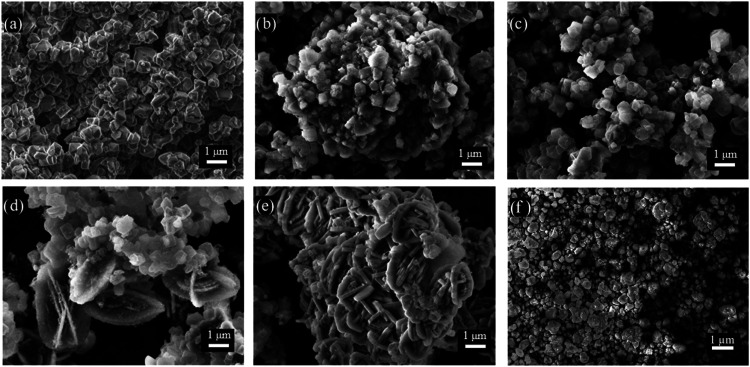
Scanning Electron Microscopy micrograph of. (a) CBV780, (b) HyZ-24, (c) HyZ-38, (d) HyZ-48, (e) HyZ-60 and (f) CBV8014.

Transmission electron microscopy (TEM) analysis (Figure 3) further supported the morphological and structural evolution, revealing important features throughout the interconversion process. The HyZ-24 sample (Figure 3a) exhibits amorphous fragments, indicating an incipient stage of interconversion. In the HyZ-38 sample (Figure 3b), increased structural organization is observed; however, the conversion becomes more evident in the HyZ-48 sample (Figure 3c), in which crystalline MFI-phase particles were identified, suggesting a more advanced stage of interconversion. The HyZ-60 sample (Figure 3d) is predominantly composed of ZSM-5 zeolite, although some structural irregularities are still present.^28^

**Figure 3:**
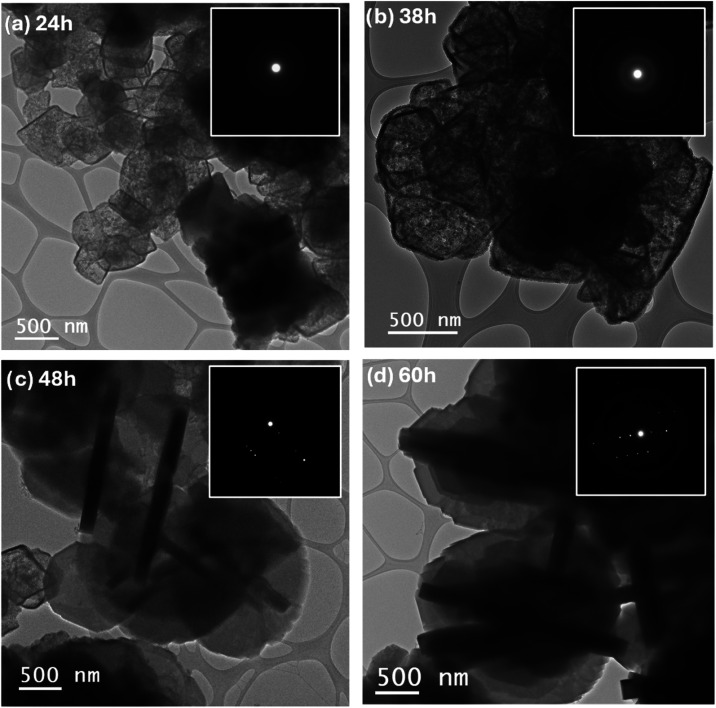
Transmission Electron Microscopy micrographs and SAED patterns shown in the insets of. (a) HyZ-24, (b) HyZ-38, (c) HyZ-48 and (d) HyZ-60.

The selected area electron diffraction (SAED) patterns shown in the insets of Figure 3a,b exhibit a diffuse halo, confirming the predominantly amorphous nature of the material, in agreement with the XRD results. In contrast, the SAED patterns in Figure 3c,d display well-defined diffraction spots, characteristic of monocrystalline structures. It is important to emphasize that while XRD provides a global assessment of crystallinity, the SAED technique is highly sensitive to local structural changes.

Figure 4a presents the ^29^Si NMR spectra of the intermediate samples, highlighting the hybrid nature of the materials. The low aluminum content of the samples (Table 1) results in the predominance of resonances associated with Q^4^(0Al) environments, characteristic of silicon atoms bonded to four other silicon atoms. The chemical shifts of the FAU and MFI structures differed significantly, with resonances centered at −108 ppm and −113 ppm, respectively.^46,47^ These variations allowed us to monitor the formation of the MFI zeolite. As the interconversion time increases, the Q^4^(0Al) signal associated with the FAU framework progressively decreases, while the resonance at −113 ppm, corresponding to the MFI structure, increases proportionally (Figure 4b). This evolution confirms the interconversion process and underscores the hybrid nature of the materials, characterized by the coexistence of resonances from both FAU and MFI zeolites.

**Figure 4:**
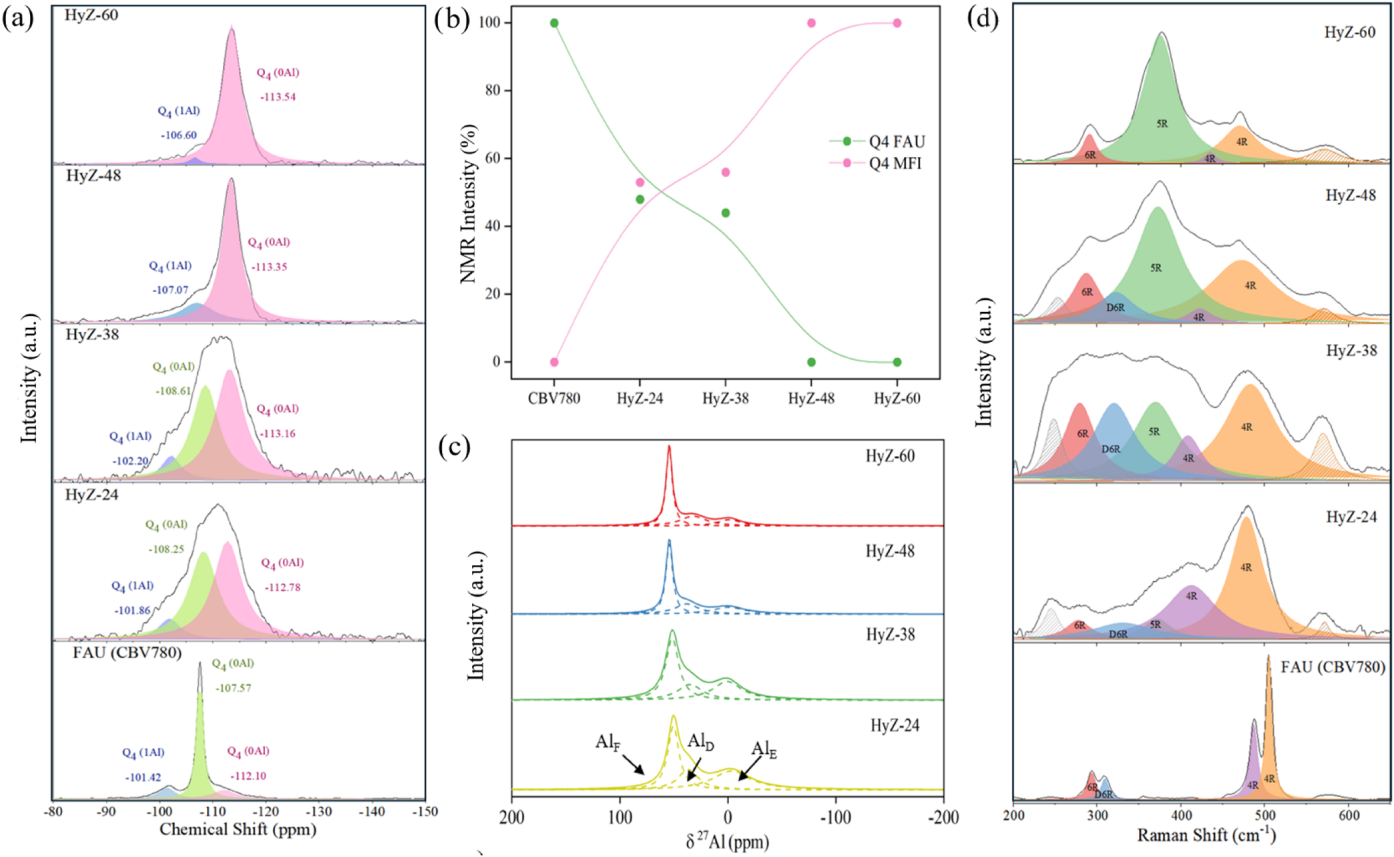
Raman and ^29^Si and ^27^Al NMR analyses of the interconverted samples. (a) ^29^Si NMR. (b) Evolution of the percentage of Q_4_ (0Al) intensity in ^29^Si NMR of the FAU and MFI zeolite. (c) ^27^Al NMR spectra. (d) Raman spectra. Each spectrum was deconvoluted to allow for the monitoring of the evolution of the various building units in the samples.

Regarding the coordination of aluminum (Figure 4c), the ^27^Al NMR analysis revealed peaks in the range of 54–61 ppm (AlF), attributed to framework aluminum incorporated into the zeolite structure; 44–45 ppm (AlD), corresponding to distorted aluminum species; and 0 ppm (AlE), associated with extra-framework aluminum located outside the main zeolite structure.^48^ A clear manifestation of the structural evolution during the interconversion process is the progressive decrease in the intensity of the peaks corresponding to distorted aluminum (AlD) and extra-framework aluminum (AlE), observed as the crystallinity of the material increases. This trend reflects a lower concentration of distorted Al species, indicating the enhanced structural ordering and greater stability of the zeolite framework. The reduction in extra-framework Al further confirms the increased incorporation of Al into the zeolite lattice, contributing to the crystallization of the final material. This incorporation had already been suggested by NH_3_-TPD data, based on the predominance of weak acid sites in the early stage samples.

Raman spectroscopy is widely recognized in literature as one of the most sensitive and effective techniques for monitoring interzeolite transformations and detecting specific structural units, even in the absence of long-range crystalline order.^33,49,50^ In line with this understanding, Raman spectroscopy (Figure 4d), supported by ^29^Si NMR results, confirmed the structural evolution from FAU to MFI zeolite. FAU exhibited characteristic bands at 300 cm^–1^ (D6R units) and 400–490 cm^–1^ (S4R units), while MFI showed a distinct band at 377 cm^–1^ (S5R units) with no D6R signals. The fully interconverted sample (HyZ-60) displayed a strong S5R band at 377 cm^–1^, minor S4R bands (435–470 cm^–1^), and an S6R band (290 cm^–1^), with no D6R band, confirming complete transformation. Intermediate samples (HyZ-24, HyZ-38, HyZ-48) showed a gradual decrease in D6R and S4R band intensities and a corresponding increase in S5R and S6R bands, reflecting progressive structural interconversion toward the MFI framework.^28,49,50^

All these results show that the interconversion of FAU to MFI zeolites proceeds through a complex sequence of structural changes. XRD and gas adsorption analyses revealed that the amorphous nonmicroporous phases of the early stages of the interconversion lack the long-range order typical of well-formed zeolites are. However, the TEM images and Raman spectroscopy indicated partial preservation of FAU structural units, suggesting that interconversion occurred gradually. SEM micrographs further confirmed the structural transition from FAU to MFI zeolite. In parallel, the Ar physisorption data showed an increase in mesoporosity in the intermediate samples, which is a key feature for improving molecular accessibility and diffusion. There is a lot of mesoporosity in the early stages of the interconversion, followed by a densification process leading to the formation of MFI zeolite. However, some of the interzeolite transformation intermediates (ITIs) uniquely combine building units of both zeolites (as evidenced by Raman spectroscopy) and large, well-defined mesoporosity, which are key features for the efficient and selective production of xylenes. As the synthesis progressed, a structural reorganization was observed, resulting in highly crystalline MFI zeolite, accompanied by changes in the acid site distribution as aluminum was progressively reincorporated into the framework. These findings provide important insights into the interzeolite conversion process and highlight the potential of microwave-assisted synthesis as an efficient strategy for developing hierarchical zeolites with tunable textural and acidic properties for advanced catalytic applications.

### 3.2 Catalytic Evaluation

The acidity and diffusion properties of the synthesized catalysts were evaluated using model reactions, including the Friedel–Crafts alkylation of benzyl alcohol with mesitylene and the cracking of triisopropylbenzene, followed by a targeted test for the sustainable production of xylenes. The conversion of 2,5-dimethylfuran (DMF) to ethanol was selected because of its potential for xylene synthesis from renewable sources,^16,17^ reducing dependence on fossil-derived feedstocks, and mitigating environmental impacts. The selectivity of this reaction is directly influenced by the balance between the acidity of the catalyst, the porous structure, which greatly determines its shape selectivity. Therefore, optimizing these parameters is essential for maximizing conversion and while minimizing undesired side reactions. The results demonstrate that the structural engineering of catalysts is a key factor in enabling more efficient and sustainable catalytic processes.

#### 3.2.1 Assessment of Acidity and Accessibility of the Catalysts

##### 3.2.1.1 Friedel–Crafts Alkylation (FC)

Friedel–Crafts alkylation of benzyl alcohol (BA) with mesitylene (ME) was selected as a test reaction to assess the accessibility and acidity of the synthesized catalysts. In FAU-type zeolites, whose micropores have a diameter of approximately 0.74 nm,^51,52^ BA can diffuse into the structure, whereas ME has limited access and is mostly confined to the external surface, thus favoring the formation of dibenzyl ether (DBE). As a result, the commercial FAU zeolite (CBV780) displayed a moderate catalytic performance, with a TOF of approximately 19.2  h^–1^ (Figure 5a), reflecting its structural characteristics. In contrast, for MFI zeolites, with micropores of approximately 0.54 nm in diameter,^28^ the reaction occurs predominantly on the external surface, as the pore openings are smaller than both reactants (BA: 0.58 nm; ME: 0.84 nm),^53,54^ hindering their diffusion into the internal framework. In this context, the TOF observed for the commercial MFI zeolite (CBV8014) (23.7  h^–1^) (Figure 5a) should not be attributed to the presence of mesoporosity, but rather to its acidity and possibly to pore mouth catalysis, which may allow partial access of the reactants to the active regions.

**Figure 5:**
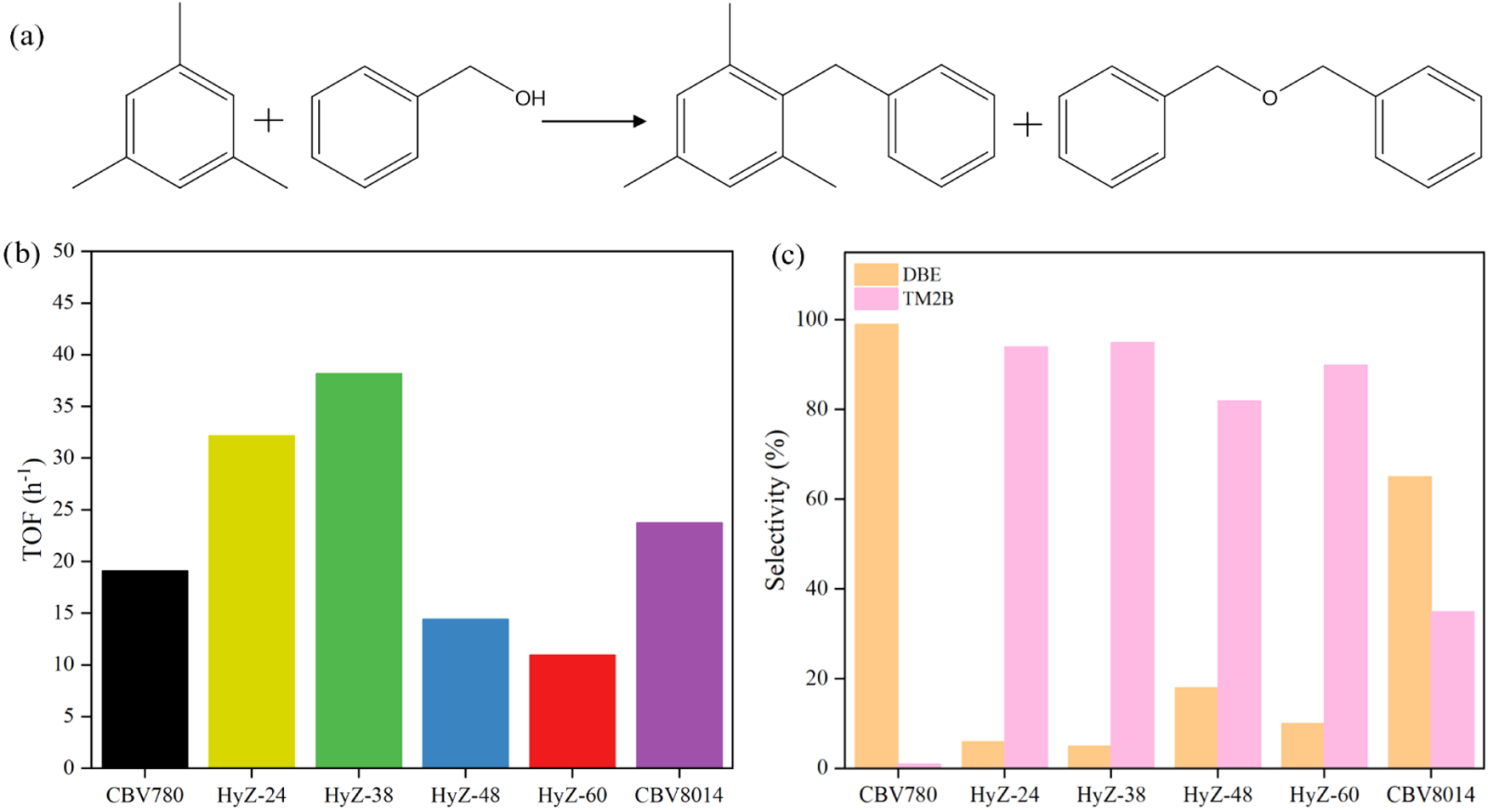
Catalytic performance of the catalyst in the Friedel–Crafts alkylation of mesitylene and benzyl alcohol. (a) Friedel–Crafts reaction scheme (b) apparent TOF; (c) selectivities to TM2B and DBE at isoconversion.

The TOF lot shows the relationship between structure and reactivity (Figure 5a). Samples HyZ-24 and HyZ-38 exhibited the highest catalytic performances, with TOF values of approximately 32.2 and 38.2 h^–1^, respectively. Physisorption data indicated that both samples contained a significant amount of mesoporosity, facilitating the diffusion of BA and ME toward internal acid sites and enhancing catalytic conversion. Starting with sample HyZ-48, a progressive decline in the TOF was observed, accompanied by an increase in the crystallinity of the MFI phase. This effect became more pronounced in HyZ-60, which exhibited the lowest TOF (10.9 h^–1^), even lower than that of the commercial MFI zeolite (CBV8014). Although both samples are microporous, the inferior catalytic performance of HyZ-60 is attributed to its lower acidity relative to the commercial MFI, the lower catalytic performance of HyZ-60 is mainly attributed to its reduced acidity compared to the commercial MFI. The normalized conversion profile of BA over time for each catalyst is available in the Supporting Material (Supporting Figure 2), allowing for a more detailed evaluation of catalytic activity.

The effects of these structural variations are also reflected in product selectivity (Figure 5b). The FAU zeolite (CBV780) favors the reaction between BA molecules due to the exclusion of ME from its micropores, resulting in nearly complete selectivity toward dibenzyl ether (DBE). In contrast, the commercial MFI zeolite (CBV8014) exhibited a more balanced selectivity between DBE and 1,3,5-trimethyl-2-benzylbenzene (TM2B). This behavior suggests that, although the micropores of MFI zeolites limit reactant diffusion, BA can still effectively access acid sites located at the pore mouths (pore mouth catalysis), allowing its conversion to DBE. Given the excess of ME and the occurrence of reactions on the external surface, the formation of the alkylated product (TM2B) is consequently favored.^29,33^

In the barely interconverted samples (i.e., HyZ24 and HyZ38), the selectivity toward 1,3,5-trimethyl-2-benzylbenzene (TM2B) increased even further, reflecting the enhanced accessibility of reactants to the pores. Textural analysis confirmed that these initial samples contained significant mesopores with diameters of approximately 4 nm, which were generated using surfactants during synthesis. The formation of mesopores enables both BA and ME to diffuse toward the internal acid sites, favoring the reaction between ME and BA and resulting in the predominance of the alkylated product.

##### 3.2.1.2 Catalytic Cracking of 1,3,5-Triisopropylbenzene (TiPBz)

To further evaluate the enhanced accessibility of the ITIs, all samples were tested for the catalytic cracking of 1,3,5-triisopropylbenzene (TiPBz) (Figure 6). This is a widely used model reaction for assessing zeolite accessibility, as TiPBz (kinetic diameter = 0.94 nm) cannot enter narrow micropores.^28,55^

**Figure 6:**
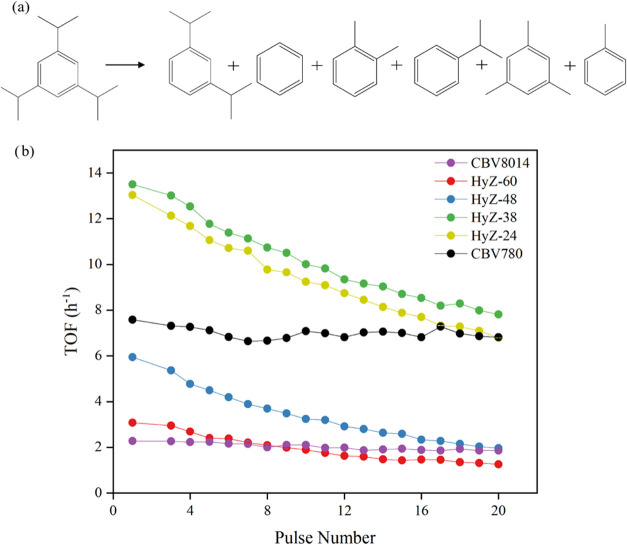
Catalytic performance of samples in the cracking of triisopropylbenzene (TiPBz): (a) Reaction scheme for TiPBz cracking, (b) apparent TOF profiles at 220 °C.

Commercial MFI zeolite, CBV8014, owing to its microporous structure with narrow channels, exhibited significantly low TOF values, as expected due to its medium pore size structure. This behavior is consistent with the known diffusion limitations of purely microporous materials, where the restricted pore size hinders the effective transport of large reactants and product molecules. In contrast, the FAU zeolite exhibited higher TOF values, which can be attributed not only to its mesoporosity, generated by postsynthetic ultrastabilization treatments, but also to its high acidity (Table 1), which provides a greater number of active sites for the catalytic cracking of bulky molecules.^28,56^

All hybrid zeolites evaluated in this study exhibited TOF values higher than those of the commercial MFI zeolite (CBV8014), demonstrating the beneficial effect of hierarchical porosity. Notably, the intermediate samples HyZ-24 and HyZ-38 showed TOF values even higher than those of the commercial FAU zeolite (CBV780), highlighting the synergistic combination of mesoporosity and preserved acidity in partially interconverted structures. This enhanced performance reinforces the ability of ITIs to bridge the gap between microporous and mesoporous materials, providing both improved molecular accessibility and high catalytic efficiency. The superior accessibility of the hybrid materials was corroborated by both Friedel–Crafts reactivity tests and detailed textural characterization.

All hybrid zeolites evaluated in this study achieved significantly higher TiPBz conversions than the commercial MFI zeolite (CBV8014). This enhanced performance is primarily attributed to their hierarchical architecture, which seamlessly integrates catalytically active micropores with mesopores to facilitate efficient mass transport.

Taken together, these findings highlight the potential of ITIs to overcome the diffusion limitations associated with conventional microporous catalysts. By enabling improved molecular transport while maintaining a high catalytic efficiency, these materials offer a robust and versatile platform for the conversion of bulky organic molecules under challenging reaction conditions.

#### 3.2.2 Evaluation for the Sustainable Production of Xylenes

The synthesis of xylenes from 2,5-dimethylfuran (DMF) and ethanol represents a promising route for producing sustainable aromatics from biomass-derived feedstocks. The process involves a sequence of reactions: catalytic dehydration of ethanol to ethylene, a Diels–Alder cycloaddition between DMF and ethylene, and a final dehydration of the oxanorbornene intermediate, leading to the formation of xylene,^38^ as illustrated in Figure 7a. The efficiency of this conversion is highly dependent on the acidic, structural, and textural properties of the catalyst.^37^ A blank test confirmed that the catalyst is essential for this reaction, as xylene yield was below 1% in the absence of catalyst. The appropriate choice of catalyst allows for maximized xylenes formation while minimizing undesired side reactions such as DMF polymerization, aromatic isomerization, and excessive cracking of intermediates.

**Figure 7:**
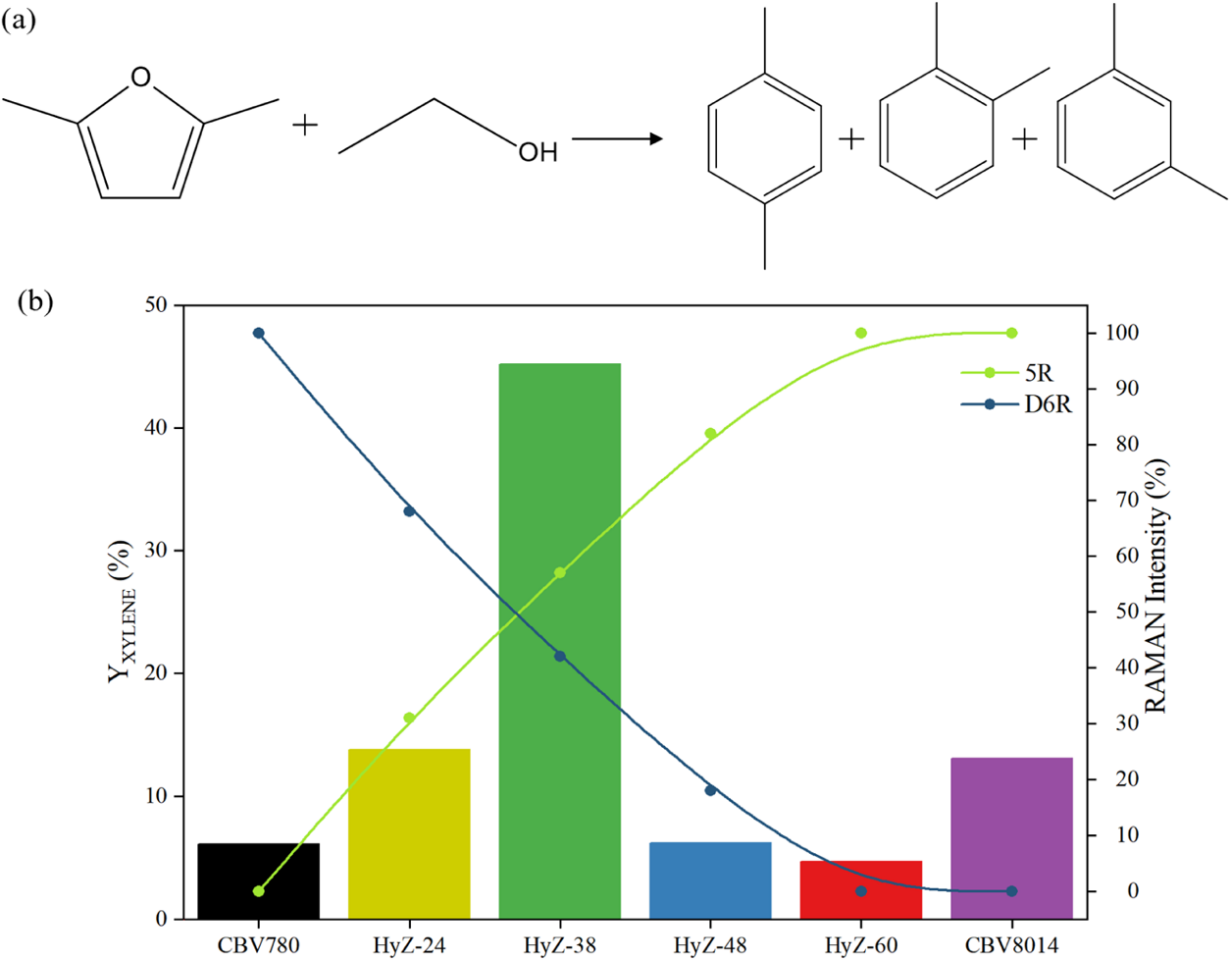
(a) Reaction scheme for the conversion of DMF and ethanol to xylenes (b) xylene yield (*Y*_XYLENE_, left axis) and normalized Raman intensities of 5R (green) and D6R (blue) units (right axis) for different hybrid catalysts.

Although the large pores and supercages of the FAU zeolite^23^ favor interactions with rather bulky molecules such as DMF and its intermediates, they do not lead to adequate selectivity toward xylene formation, resulting in unsatisfactory yields. However, although these characteristics favor interactions with molecules such as DMF and its intermediates, they are not accompanied by adequate selectivity toward xylene formation, resulting in unsatisfactory yields. This behavior is illustrated in Figure 7b, where the CBV780 sample shows a low xylene yield, indicating the occurrence of multiple parallel reactions. The lack of effective spatial constraints in the FAU framework allows for the formation of undesirable byproducts such as DMF dimers, overalkylated species, and condensation intermediates. These products are favored by the prolonged residence time of reactive intermediates at the acid sites, which increases the likelihood of polymerization and condensation reactions.

In contrast, the MFI structure (CBV8014), with its smaller pores (∼0.47 nm), imposes spatial constraints that restrict the formation of overalkylated products and suppress undesired rearrangements. However, this selectivity may also hinder diffusion of the oxanorbornene intermediate, thereby reducing xylene formation. This effect is illustrated in Figure 7b, where CBV8014 exhibits a low xylene yield, confirming that the diffusion barriers negatively impact its catalytic performance.

Thus, the hybrid catalysts developed in this study, which feature building units from both FAU and MFI zeolites, combining the high accessibility of the former with the shape selectivity of the latter represent a good balance between efficient diffusion and steric control, merging the high accessibility of FAU with the shape selectivity of MFI. This unique combination is expected to enhance catalytic activity while improving the selective formation of xylenes

In fact, the HyZ-38 catalyst exhibited the highest xylene yield (45.2%) (Figure 7b), indicating that it was the most efficient material for converting DMF and ethanol into xylene. This performance can be attributed to its hybrid nature, as evidenced by Raman spectroscopy, which confirmed the structural unit characteristics of both the FAU and MFI frameworks. This structural duality is also visible in Figure 7b through the simultaneous distribution of D6R units, typical of FAU and 5R units associated with MFI, reinforcing the integration of both topologies in the hybrid material. Notably, the sample that exhibited the highest relative intensity of these two units, indicating a more balanced degree of hybridization, also achieved the highest xylene yield. This performance surpassed even that of the physical mixture of commercial zeolites FAU (CBV780) and MFI (CBV8014), prepared with a 50% acid site contribution from each component and an overall acidity equivalent to that of the hybrid catalyst HyZ-38 (Supporting Figure 3). This result reinforces the direct correlation between the well-distributed hybrid structure and the optimized catalytic performance.

This hybrid structure balances the accessibility and shape selectivity, favoring the formation of xylenes. The well-distributed mesoporosity enhances the diffusion of reactants and products, whereas the MFI structural units contribute to shape selectivity by restricting the formation of secondary products. Additionally, the balanced distribution of strong and weak acid sites provides optimal catalytic conditions, maximizing conversion while minimizing side reactions. Strong acid sites play a key role in activating the reactant molecules and driving the main steps of catalytic conversion, whereas weak acid sites help suppress undesired consecutive reactions that could compromise selectivity. This synergistic combination of acid strength and site distribution resulting from the controlled degree of interconversion between the FAU and MFI phases is essential for achieving high yields and selectivity in the production of xylenes from renewable sources.

As expected, the HyZ-48 and HyZ-60 catalysts showed the lowest xylene yields (6.2% and 4.7%, respectively) because of their complete interconversion into the MFI structure. The predominance of microporosity in these materials reduces the access to active sites and hinders product diffusion, limiting the release of reaction intermediates. This behavior was confirmed in the model reaction tests, in which these materials exhibited the typical characteristics of MFI zeolites, demonstrating that the diffusion constraints imposed by this structure negatively affect the reaction yield.

Despite its high mesoporosity and acidity profile being similar to that of the HyZ-38 catalyst, the HyZ-24 catalyst did not exhibit a high xylene yield, demonstrating that these factors alone are insufficient to optimize the conversion. Raman analysis showed that this material contained significantly fewer MFI structural units than HyZ-38, hindering the shape selectivity required to promote xylene formation. Thus, although HyZ-24 offers good accessibility to reactants, the lack of an MFI framework limits its performance, resulting in a lower yield.

These results demonstrate that a balanced combination of FAU and MFI structural units is essential for optimizing catalytic efficiency and ensuring accessibility without compromising selectivity, thus explaining the superior performance of HyZ-38 compared to the other evaluated materials. Our findings highlight that maximizing the xylene yield requires precise tuning of the catalyst structure, revealing that the partial interconversion of zeolite introduced elsewhere is an effective strategy to overcome the limitations of conventional zeolites while enabling the selective production desirable products, in this case aromatics from renewable sources.

Finally, when comparing our results with the available literature, it becomes clear that the hierarchical hybrid catalysts developed in this work represent a promising and distinctive alternative for renewable xylene production. Previous studies have shown that *p*-xylene can be produced from the reaction between DMF and ethanol, with yields ranging from 67 to 79% using modified ZSM-5 zeolites.^17,57^ Despite these advances, those studies still rely on conventional synthesis routes, which are associated with long preparation times, high energy consumption, and limited control over the generation of hierarchical structures, factors that restrict scalability and hinder alignment with the principles of sustainable chemistry. Other works have reported even higher *p*-xylene yields, reaching or exceeding 90%, by using pure ethylene in combination with β or H-β zeolite-based catalysts.^58^ However, these processes require an external ethylene supply, often derived from fossil resources, as well as additional separation and purification steps, which increase operational complexity and reduce the overall sustainability of the route. In contrast, our approach combines the direct use of DMF and ethanol with hybrid catalysts obtained through microwave-assisted FAU-to-MFI interconversion, providing a more sustainable, energy-efficient, and integrated process. It is worth noting that the primary objective of this study was to compare the performance of these hybrid materials with that of conventional zeolites to assess the actual benefits of hierarchical interconversion. Reaction conditions were not yet optimized, further highlighting the potential of this approach to achieve even higher yields in future studies, without compromising the sustainability and energy efficiency of the process.

## 4 Conclusions

In this study, Interzeolite Transformation Intermediates (ITIs) were synthesized via microwave-assisted interconversion of FAU to MFI phases and evaluated for the conversion of 2,5-dimethylfuran (DMF) and ethanol to xylenes for sustainable aromatics production from renewable sources. The catalysts were systematically characterized using XRD, SEM, TEM, NMR, Raman spectroscopy, and NH_3_-TPD, showing that the ITIs combine structural features of both the parent and daughter zeolites and exhibit well-developed mesoporosity and strong acidity. The optimized HyZ-38 catalyst, which retained both FAU and MFI structural units, achieved a superior xylene yield of 45.2%, which was attributed to the synergistic balance between mesoporosity and shape selectivity, which enhanced diffusion while minimizing side reactions, as confirmed in model catalytic tests, including Friedel–Crafts alkylation and triisopropylbenzene cracking. Microwave-assisted interzeolite transformation significantly reduced synthesis time and energy consumption, contributing to a more sustainable catalyst preparation. The results highlight interzeolite transformation as an effective strategy for designing advanced catalysts tailored for biomass valorization and renewable aromatics production.

## Supplementary Material



## Notes

GC calibration chromatogram for the reaction of 2,5-dimethylfuran with ethanol; normalized conversion of benzyl alcohol (BA) over time; hydrofluoric acid (HF) safety protocol (PDF)
