# Supplementary material for: Combining Mesoporosity and Shape Selectivity in FAU–MFI Interzeolite Intermediates for Renewable Xylenes Production
Source: ACS Sustain Chem Eng. 2025 Aug 19;14(1):72–85. doi: 10.1021/acssuschemeng.5c05295 (PMC12801384; doi:10.1021/acssuschemeng.5c05295)
Supplement: Supplementary file 1 [file sc5c05295_si_001.pdf]

Supporting Information

Combining Mesoporosity and Shape Selectivity in FAU–MFI Interzeolite Intermediates for Renewable Xylenes Production

Daniela O. Campos[a,b], Anna D. F. F. Monsores[a,b], Natasha M. Suguihiro[b,e], Donato A. G. Aranda[a,b], Javier Garcia Martinez[c], Pedro N. Romano[b,d,e] and João M.A.R. de Almeida[b,f,*]

[a] Escola de Química, Universidade Federal do Rio de Janeiro, Av. Athos da Silveira Ramos, 149, Rio de Janeiro, Brazil, 21941-909.

[b] LIPCAT/IDlab (Laboratório de Intensificação de Processos e Catálise), Universidade Federal do Rio de Janeiro (UFRJ), Rio de Janeiro 21941-594, RJ, Brazil.

[c] Laboratorio de Nanotecnología Molecular, Departamento de Química Inorgánica, Universidad de Alicante, 03690, Alicante, Spain. ORCID:0000-0002-7089-4973

[d] Nanotechnology Engineering Program, Alberto Luiz Coimbra Institute for Graduate Studies and Research in Engineering (COPPE), Federal University of Rio de Janeiro, Avenida Horacio Macedo, 2030 21941-972, Rio de Janeiro, RJ, Brazil

[e] Campus Duque de Caxias, Universidade Federal do Rio de Janeiro, Rodovia Washington Luiz, 19593, Rio de Janeiro, Brazil, 25240-005.

[f] Instituto de Química, Universidade Federal do Rio de Janeiro, Av. Athos da Silveira Ramos, 149, Rio de Janeiro, Brazil, 21941-909.

*j.monnerat@iq.ufrj.br (J.M.A.R. de Almeida)

The retention times (RT) of all compounds were identified by calibration using the reactants (2,5-dimethylfuran, ethanol, and n-heptane) and the products of interest (xylene mixture).

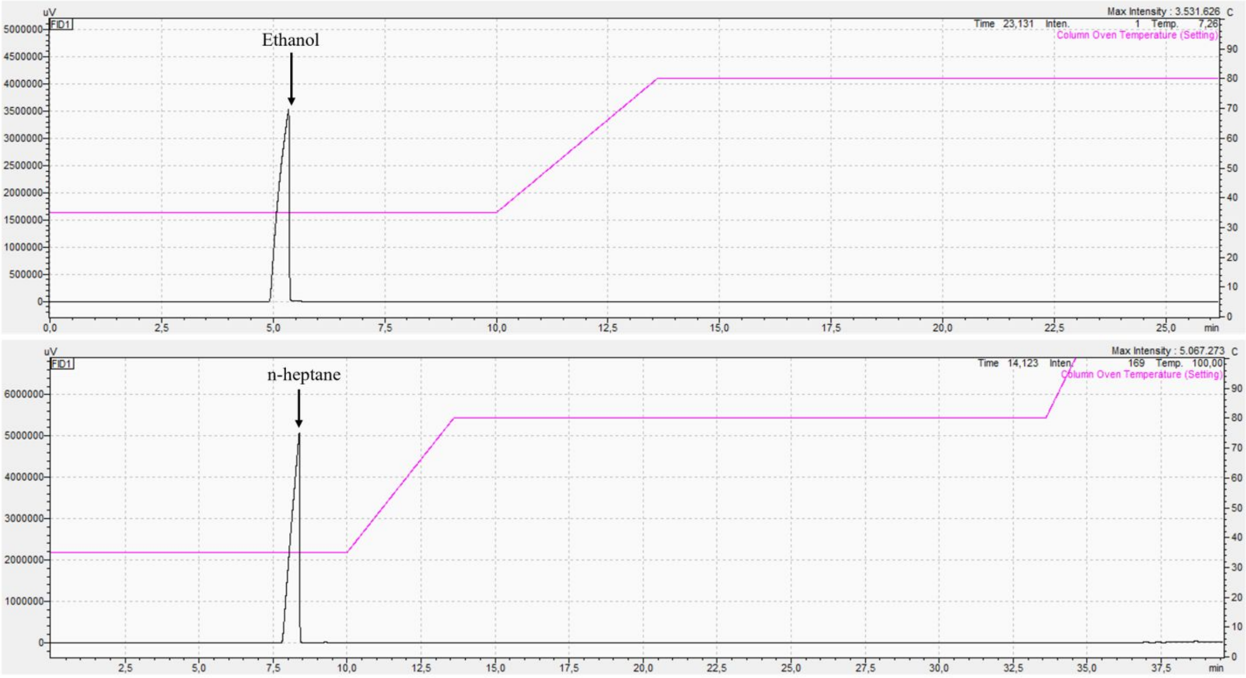

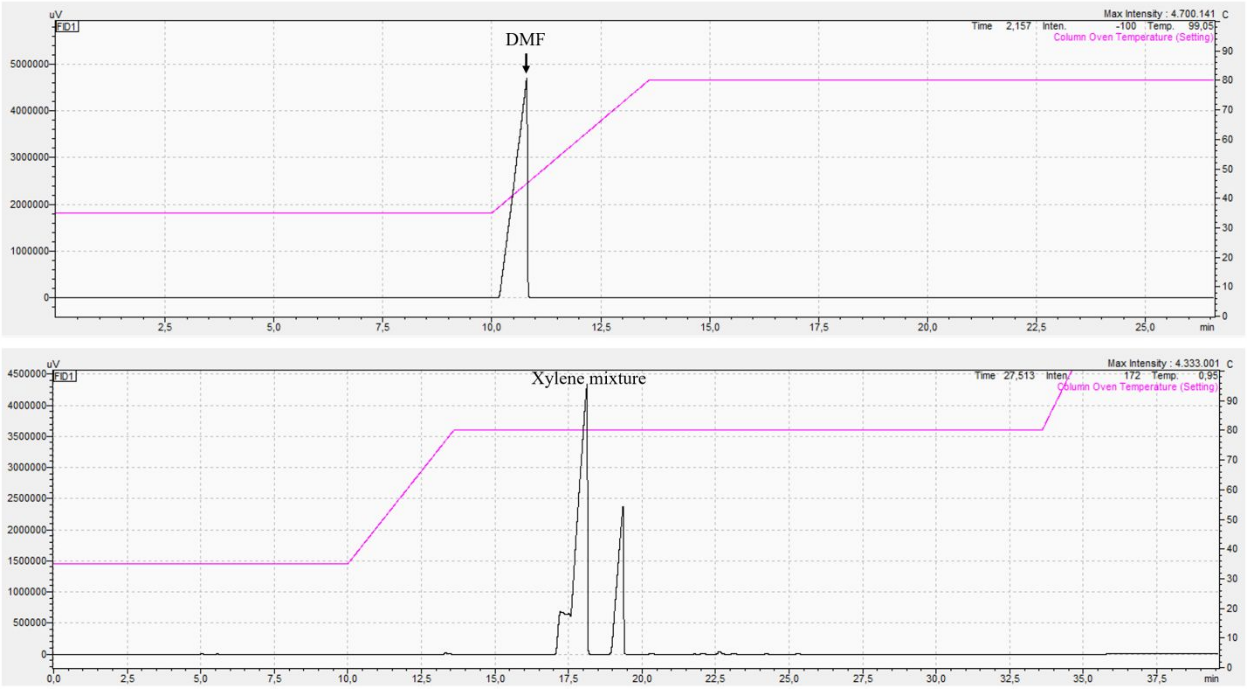

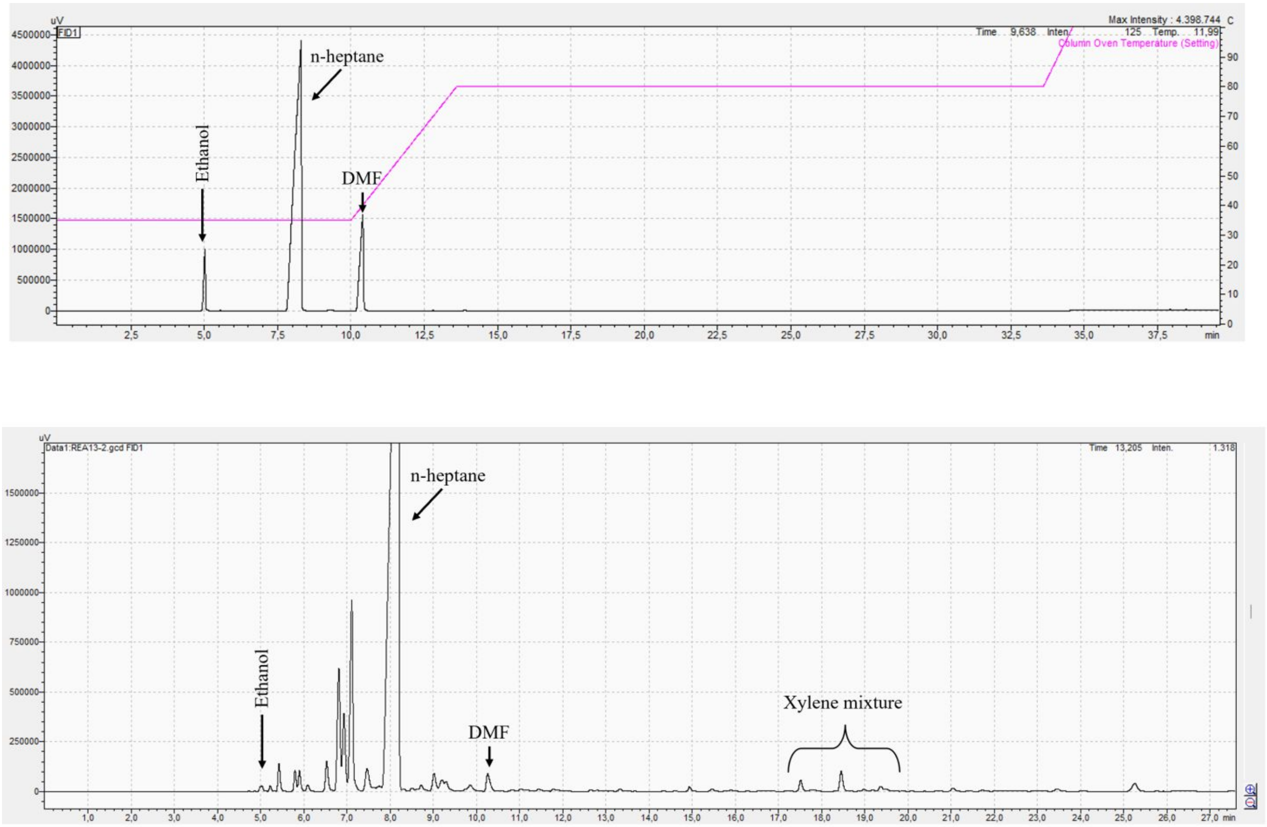

Supplementary Figure 1 - GC calibration chromatogram for the reaction of 2,5-dimethylfuran with ethanol. Standards of 2,5-dimethylfuran, ethanol, n-heptane, and xylene mixture were used.

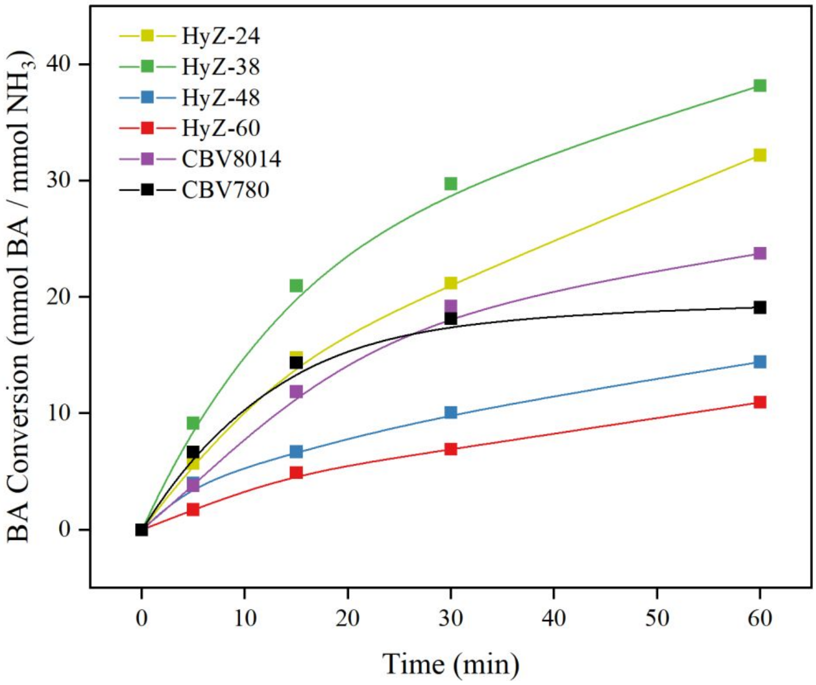

Supplementary Figure 2 - Normalized conversion of benzyl alcohol (BA) over time, based on the amount of acid sites (mmol of NH_3), for different zeolite catalysts. Reactions were conducted at 120 °C.

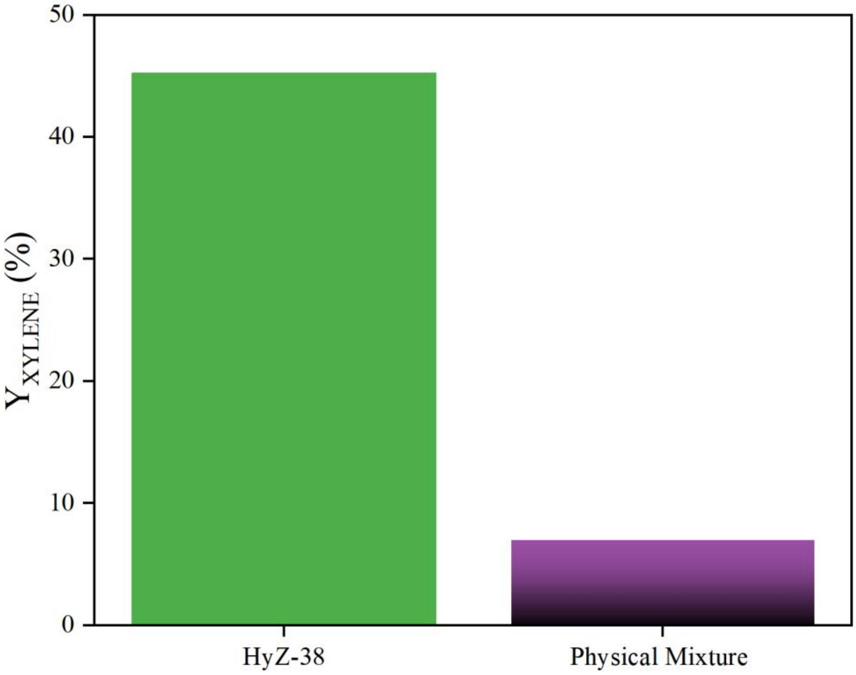

Supplementary Figure 3 – Comparison of xylene yield obtained with the HyZ-38 catalyst and a physical mixture of commercial zeolites FAU (CBV780) and MFI (CBV8014). The mixture was formulated so that each zeolite contributed 50% of the total acidity, resulting in an overall acid site content equivalent to that of HyZ-38 (39.3 μmol NH_3).

Hydrofluoric acid (HF) safety protocol: Due to the high toxicity and corrosive nature of hydrofluoric acid, all procedures involving HF were conducted under strict safety measures. The acid was handled exclusively in a certified fume hood, and all personnel used appropriate personal protective equipment (PPE), including nitrile gloves, a face shield, lab coat, chemical-resistant apron, and a respirator with an acid vapor filter. Microwave digestion procedures were performed in closed TFM vessels compatible with HF, using a high-pressure microwave digestion system equipped with automated safety controls. All researchers involved received specific training in HF handling, and calcium gluconate gel was always kept accessible as an emergency antidote in case of accidental skin exposure. These safety protocols were rigorously followed throughout experimental work.
